# The hypothalamo-pituitary-adrenal (HPA) axis in sheep is attenuated during lactation in response to psychosocial and predator stress

**DOI:** 10.1016/j.domaniend.2015.11.003

**Published:** 2016-04

**Authors:** C.R. Ralph, A.J. Tilbrook

**Affiliations:** a Division of Livestock and Farming Systems, South Australian Research and Development Institute, The University of Adelaide, Roseworthy, South Australia, 5371, Australia; b Department of Physiology, Monash University, Melbourne, Victoria, 3800, Australia

**Keywords:** Stress, HPA axis, Oxytocin, Lactation, Hyporesponsiveness

## Abstract

Activation of the hypothalamo-pituitary-adrenal (HPA) axis by psychosocial stress is attenuated during lactation. We tested the hypothesis that lactating ewes will have attenuated HPA axis responses to isolation and restraint but will have greater responses to predator stress in the form of barking dogs. We imposed two 4 h stressors: psychosocial stress (isolation and restraint of ewes) and predator stress (barking dogs). Blood was collected intravenous every 10 min from nonlactating ewes (n = 6), lactating ewes with lambs present but not able to be suckled (n = 6), and lactating ewes with lambs present and able to be suckled (n = 6). Plasma cortisol and oxytocin were measured. For nonlactating ewes, cortisol increased (*P* < 0.01) in response to both stressors, and these increases were greater (*P* < 0.01) than that in the lactating animals. For lactating ewes with lambs present but unable to be suckled, cortisol increased (*P* < 0.05) in response to both stressors with a greater response to barking dogs (*P* < 0.05). For lactating ewes with lambs present and able to be suckled, cortisol increased (*P* < 0.01) in response to barking dogs only. Plasma oxytocin was greater (*P* < 0.01) in lactating ewes than in nonlactating ewes and did not change in response to the stressors. In conclusion, lactating ewes are likely to have a greater HPA axis response to a stressor that may be perceived to threaten the welfare of themselves and/or their offspring. The role of oxytocin in attenuation of the HPA axis to stress in sheep is unclear from the current research and requires further investigation.

## Highlights

• The response of the hypothalamo-pituitary-adrenal axis to stress is attenuated during lactation in sheep.
• The extent of this attenuation is influenced by the opportunity to suckle lambs and the type of stressor.
• Stressors that pose an immediate threat to the offspring evoke greater stress responses.

## 1 Introduction

Activation of the hypothalamo-pituitary-adrenal (HPA) axis by psychosocial stress is commonly attenuated during mid–late pregnancy and lactation in a range of species, including mice [1], rats [2], humans [3], and sheep [4]. It has been suggested that this natural state of stress hyporesponsiveness in lactating females is important for the wellbeing and mental health of the dam, [5] which, in turn, contributes to her maternal behavior and ability to safely rear the offspring. Although this makes sense, there are clearly conditions where it would be beneficial for the lactating female to elicit stress responses to stimulate behavioral and physiological actions designed to protect the offspring in a threatening environment. This is in keeping with a biological role of stress responses to maintain homeostasis and promote survival [6], [7]. The hypothesis that lactating females will have stress responses when their offspring are threatened has not been formally tested although there is indirect evidence for this in rats [8], rhesus monkeys [9], and humans [10]. For example, in humans, breastfeeding mothers showed stress-induced hormonal, autonomic, and psychological responses to a single breath of 35% carbon dioxide, and it was suggested that a full stress response by the mother was required because this challenge would be perceived as a threat to her survival and, in turn, the survival of her infant [10]. In our previous studies of HPA axis hyporesponsiveness in lactating ewes, the stressor was isolation of the ewe and her lamb from other flock mate ewes and restraint of the ewe in a pen [4]. The lamb was present and was not restrained. It is unlikely that there was perception by the ewes that their lambs would have been threatened in this environment. In contrast, stress in the form of barking dogs, which could be considered a predator stress, would likely be perceived as a threat to the lamb and, under this circumstance, elicit a stress response. Indeed, when exposed to a barking dog, lactating ewes have shown an increase in plasma cortisol although lower than nonlactating ewes [4].

The mechanisms for attenuated stress responses in late pregnant and lactating females to many stressors are complex and multifaceted (for review see [5]). We showed that the presence of the offspring and sucking by the offspring are important in attenuating HPA axis responses to isolation and restraint in lactating ewes [4]. The mechanisms by which the presence of the lambs, and suckling, influence the activity of the HPA axis are unknown, but at least part of the mechanism may involve oxytocin, which is released in response to cues from the infant, especially sucking [5], [11], [12], [13]. There is experimental evidence that oxytocin can act centrally to attenuate the activity of the HPA axis [for review see [14]], and we showed in the sheep that oxytocinergic neurons are located in close proximity to corticotrophin releasing hormone (CRH) and arginine vasopressin (AVP) neurons in the paraventricular nucleus (PVN) [4]. If oxytocin is involved in the attenuation of the stress-induced activity of the HPA axis during lactation, this might be reflected in different concentrations of circulating oxytocin in lactating ewes exposed to stressors that are perceived to threaten the lamb and those that are not.

In this study, we tested the hypothesis that lactating ewes will have attenuated cortisol responses to isolation and restraint but will have greater responses to predator stress in the form of barking dogs. To establish the importance of suckling (and oxytocin) in influencing these responses, we compared plasma oxytocin and cortisol to restraint and predator stress in nonlactating control ewes to lactating ewes with lambs present and unable to suck and lambs present and able to suck.

## 2 Materials and methods

All animal procedures were conducted with prior institutional ethical approval under the requirements of the Australian Prevention of Cruelty to Animals Act 1986 and the National Health and Medical Research Council/Commonwealth Industrial Research Organization/Australian Research Council Code of Practice for the Care and Use of Animals for Scientific Purposes.

### 2.1 Animals

The study used 18 Australian Merino ewes and was conducted during the nonbreeding season at the Monash Large Animal Facility, at Werribee, Victoria, Australia (38°S). The 18 ewes consisted of 6 that were not lactating (nonlactating) and 12 that were lactating. The ewes were chosen randomly from a flock of 200 ewes to which rams had been introduced for 8 wk during the normal breeding season. The ewes were undergoing natural estrous cycles during this time. Lactating ewes were randomly selected from ewes that gave birth to a single lamb, and the experiment was conducted when the lambs were 6 wk old. Nonlactating ewes were flock mates that failed to lamb.

All sheep were housed in adjacent individual pens (0.5 × 1.2 m) for 1 wk before the experiment. Indwelling catheters (Dwellcath; Tuta Laboratories, Lane Cove, Australia) were placed in 1 jugular vein of each ewe the day before sampling and treatment. The jugular vein was located through palpation of the neck. The catheter inserted directly through the skin into the jugular vein, verified by the presence of venous blood in the catheter. A single suture was used to fix the catheter to the skin. Patency of each catheter was maintained by flushing with 5-mL heparinized saline (0.5%) at each time of sampling. The catheter was removed after each sampling period.

### 2.2 Experimental design

The ewes were divided into the following groups (n = 6 ewes per group): (1) nonlactating ewes, (2) lactating ewes with lambs present but unable to be suckled, and (3) lactating ewes with lambs present and able to be suckled. For lactating ewes with lambs present but unable to be suckled, the lambs were present in the pens with their mothers. A partition prevented suckling but allowed auditory, olfactory, and limited tactile stimulation between the lamb and mother [4]. For lactating ewes with lambs able to be suckled, the lambs were present in the pens with their mothers and were able to move about freely and suck without restriction.

Animals were exposed to stress in random order over 2 d, with blood samples for individual animals collected over 8 h. The second stress was imposed after a 14-d rest period. Different stressors were imposed on all animals on each of the experimental days. One stressor was isolation and restraint stress that was imposed for 4 h. The other consisted of 3 dogs barking for 5 min continuously, that was imposed every hour on the hour for 4 h.

On each experimental day, blood samples (5 mL) were collected every 10 min for 8 h. After 4 h of sampling, the stressor was imposed for the remaining 4 h. Isolation and restraint stress was imposed as previously described [15]. Briefly, each ewe was moved to a novel pen of the same dimensions that contained no sheep on either side or in front or behind. Olfactory and auditory stimuli were limited, and visual stimuli were removed. The lambs of lactating ewes were transferred to the novel pen with their mothers. Each ewe was fitted with a harness that was used to restrain it to the side of the pen, and the pen was completely enclosed on all sides and top by hessian (RN and G Lowin, Fitzroy, Australia). Once a ewe was restrained only the head could be moved freely. The animals had access to water ad libitum. On the experimental day, animals were not fed until the conclusion of the 8-h sampling; this was to avoid any confounding effects of feed. The lambs of lactating ewes were not restrained. For the barking dogs stress, 3 dogs were introduced to the experimental shed immediately after the blood sample collected at 4 h and barked continuously for 5 min. This was repeated each hour for 4 h. The dogs were visible to all sheep, but physical contact was prevented. We have shown previously that this stressor reliably activates the HPA axis in nonlactating ewes [16].

Blood was held on ice for up to 10 min, then, plasma was harvested from the blood by centrifugation at 4°C for 10 min at 3000 rpm (24,149*g*). Plasma was decanted immediately after centrifugation and stored at −20°C until assay. Plasma concentrations of cortisol and oxytocin were assayed.

### 2.3 Radioimmunoassays

Plasma cortisol was measured with an extracted radioimmunoassay [17] using hydrocortisone (H-4001; Sigma Chemical Company, St Louis, MO, USA) as standard. The assay used [3H]-cortisol (Amersham Pharmacia Biotech, UK, Buckinghamshire HP, England) as tracer and a dichloromethane extracion procedure with a mean (± standard error of the mean [SEM]) recovery of 93.2 ± 2.8%. There were 8 assays conducted, and the sensitivity ranged from 0.15 to 0.47 ng/mL with a mean of 0.33 ng/mL. The mean intra-assay coefficient of variation was 7.81%. The mean interassay coefficient of variation was 12.06%.

Oxytocin in plasma was measured using a Phoenix Pharmaceuticals Oxytocin Radioimmunoassay Kit (Belmont, CA, USA) following a similar procedure to that described by Marazziti [18]. There was a 100% cross-reactivity with oxytocin and no cross-reactivity with AVP. Nine assays were conducted and the sensitivity ranged from 0.3 to 2.3 pg/mL with a mean of 1.2 pg/mL. The mean intra-assay coefficient of variation was 5.22%, and the mean interassay coefficient of variation was 8.01%.

### 2.4 Statistical analysis

Normality of the data sets was tested using the Kolmogorov–Smirnov statistic, and homogeneity of variance was tested using Levene's test. No transformations were necessary.

Repeated measures analysis of variance was used to compare the plasma concentrations of cortisol and oxytocin within and between groups and stressors. The within subjects factors were stress (isolation and restraint or barking dogs) and the sampling times (time). The between subjects factor was group (nonlactating ewes, lactating ewes with lambs present but unable to be suckled, and lactating ewes with lambs present and able to be suckled). Individual animals were also included as factors. Three analyses were conducted. One was on all data, and another was on mean concentrations. The third analysis was on proportional increase in plasma concentrations in response to stress. The analysis on all data allowed comparisons over time, that is within subjects, and overall between subjects. Analysis on the mean concentrations allowed for comparison between pre- and post-treatment. The key comparison to test the hypothesis that “lactating ewes will have attenuated cortisol responses to isolation and restraint but will have greater responses to predator stress in the form of barking dogs” is the cortisol responses in the lactating ewes when subjected to isolation and restraint relative to barking dogs. These are the cortisol responses over time and the concentrations of cortisol between these treatments. To establish the importance of suckling in influencing these responses, the key comparison is plasma cortisol to both stressors in lactating ewes with lambs present and unable to suck and lambs present and able to suck. Comparisons were also made between nonlactating ewes and lactating ewes to confirm that lactation attenuates HPA axis responses to stress.

**Fig. 1:**
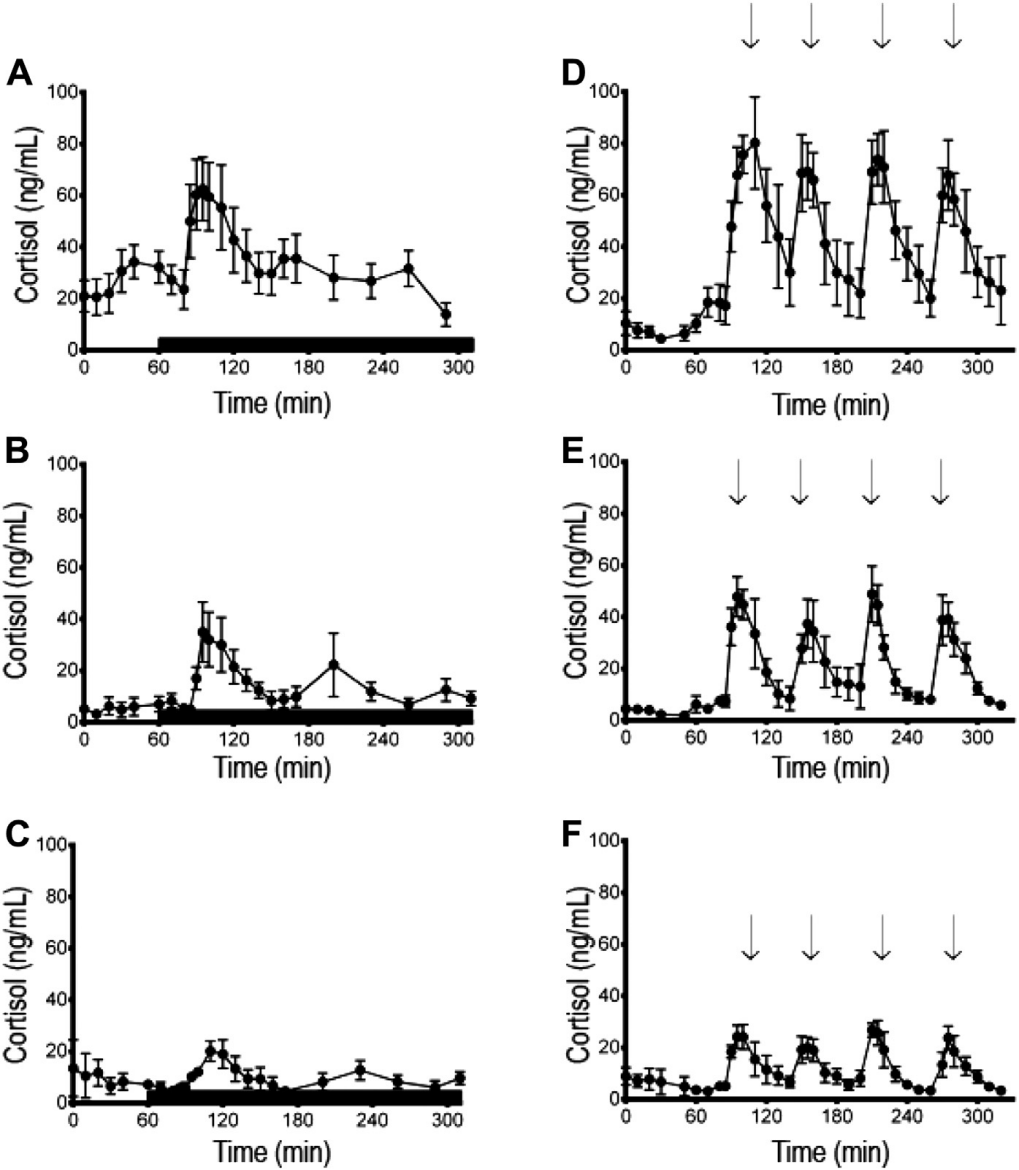
Mean (± standard error of the mean) cortisol concentration before and during isolation and restraint stress or exposure to a barking dog. (A) Nonlactating ewes exposed to isolation and restraint stress; (B) lactating ewes with lambs present but unable to suckle exposed to isolation and restraint stress; (C) lactating ewes with lambs present and able to suckle exposed to isolation and restraint stress; (D) nonlactating ewes exposed to barking dogs; (E) lactating ewes with lambs present but unable to suckle exposed to barking dogs; and (F) lactating ewes with lambs present and able to suckle exposed to barking dogs. Solid bar indicates isolation and restraint stress (A, B, and C), and arrows indicate exposure to barking dogs (D, E, and F). Comparisons between lactating ewes exposed to isolation and restraint (B and C) and lactating ewes exposed to barking dogs (E and F) tested the hypothesis that lactating ewes will have attenuated cortisol responses to isolation and restraint but will have greater responses to predator stress in the form of barking dogs. Comparisons between cortisol to both stressors in lactating ewes with lambs present and unable to suck (B and E) and lambs present and able to suck (C and F) establish the importance of suckling in influencing these cortisol responses. Comparisons between nonlactating ewes (A and D) and lactating ewes (B and E, C and F) confirm the effect of lactation to attenuate the responsiveness of the hypothalamo-pituitary axis to stress.

**Fig. 2:**
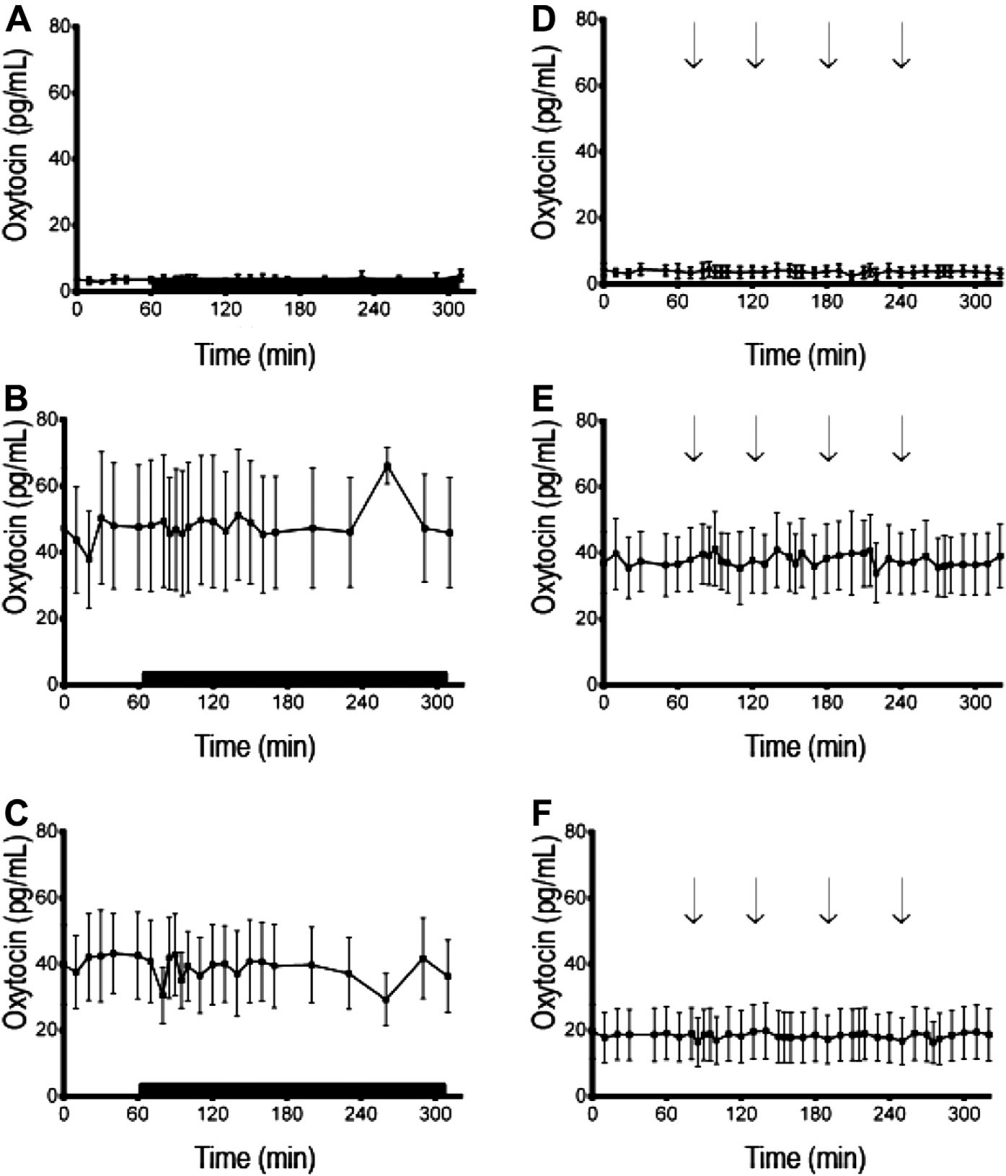
Mean (± standard error of the mean) oxytocin concentration before and during isolation and restraint stress or exposure to a barking dog. (A) Nonlactating ewes exposed to isolation and restraint stress; (B) lactating ewes with lambs present but unable to suckle exposed to isolation and restraint stress; (C) lactating ewes with lambs present and able to suckle exposed to isolation and restraint stress; (D) nonlactating ewes exposed to barking dogs; (E) lactating ewes with lambs present but unable to suckle exposed to barking dogs; (F) lactating ewes with lambs present and able to suckle exposed to barking dogs. Solid bar indicates isolation and restraint stress (A, B, and C), and arrows indicate exposure to barking dogs (D, E, and F).

Because it is well appreciated that there can be activation of the HPA axis in ewes at the commencement of sampling [15], the analysis on each experimental day was broken down such that the major comparison occurred between 1 h before imposition of the stressor (termed prestress), when the HPA axis activity was basal, and the 4 h after imposition of the stressor (termed stress). We have validated this approach previously [19]. The complete data set was illustrated in Figure 1, and the mean concentrations and proportional change in these concentrations are illustrated in Table 1. Results from the analysis of all oxytocin data are presented Figure 2 and the mean (±SEM) concentrations of oxytocin are given in the text.

## 3 Results

### 3.1 Cortisol

Mean overall plasma concentrations of cortisol differed between groups and were greater (*P* < 0.001) in nonlactating ewes than both groups of lactating ewes, irrespective of the stage of the experiment (Fig. 1). There was also an overall within subjects effect of time (*P* < 0.001).

In nonlactating ewes, the plasma concentrations of cortisol increased (*P* < 0.01) from the pre-treatment period to the period of isolation and restraint stress (Fig. 1A). On the day of introduction of barking dogs, the plasma concentrations of cortisol did not change during the pre-treatment period, and there was an increase (*P* < 0.001) in plasma cortisol with each introduction of the barking dogs (Fig. 1D). There was a time × stress interaction because of greater increases in cortisol in response to the barking dogs than isolation and restraint stress (*P* = 0.01; Table 1).

In lactating ewes with lambs present but unable to be suckled, there was an effect of time for both stressors (*P* = 0.001). On the day of isolation and restraint stress, there was an increase (*P* < 0.01) in plasma cortisol during isolation and restraint (Fig. 1B). For the barking dogs, plasma concentrations of cortisol increased (*P* < 0.01) with each introduction of the barking dogs returning to baseline within 30 to40 min (Fig. 1E). The increase in plasma concentrations of cortisol during the period of introduction of the barking dogs was greater (*P* < 0.05) than during the period of isolation and restraint (Table 1).

In lactating ewes with lambs present and able to be suckled, there was no overall effect of time (*P* = 0.07), but there was a time × stress interaction (*P* = 0.008). Further partitioning of the analysis revealed that plasma cortisol did not vary throughout the day of isolation and restraint stress (Fig. 1C), whereas there were increases in cortisol in response to introduction of the barking dogs (*P* < 0.01; Fig. 1F). Therefore, the proportional increase in plasma concentrations of cortisol in lactating ewes with lambs present and able to be suckled was greater (*P* < 0.05) in response to barking dogs than isolation and restraint (Table 1).

During the periods of stress, the plasma concentrations of cortisol in the nonlactating ewes were greater (*P* < 0.01) than that in both the lactating ewes with lambs present but unable to be suckled and lactating ewes with lambs present and able to be suckled (Table 1, Fig. 1). The plasma concentrations of cortisol during both isolation and restraint stress and the barking dogs stress were greater (*P* < 0.01) in lactating ewes with lambs present but unable to be suckled than that in lactating ewes with lambs present and able to be suckled (Table 1, Fig. 1).

### 3.2 Oxytocin

Differences were not detected in overall plasma concentrations of oxytocin among lactating ewes regardless of ability of lambs to suck. Nevertheless, there was a between groups effect (*P* = 0.008) with the mean (±SEM) plasma concentrations of oxytocin (ng/mL) being greater in lactating ewes (1.5 ± 0.02) than that in the nonlactating ewes (0.8 ± 0.02).

There were no consistent effects of time on plasma concentrations of oxytocin for any of the groups with no discernible difference between the pre-treatment period and the period of stress. For the nonlactating ewes, there was also no difference in plasma concentrations of oxytocin on the day of isolation and restraint stress and the day of barking dogs (Fig. 2). In contrast, the plasma concentrations of oxytocin were greater on the day of isolation and restraint stress than that on the day of barking dogs for both the lactating ewes with lambs present but unable to be suckled (*P* = 0.002) and the lactating ewes with lambs present and able to be suckled (*P* = 0.047).

## 4 Discussion

It is clear from these results that the responsiveness of the HPA axis to stress in ewes is attenuated during lactation and that the extent of this attenuation is influenced by the type of stressor. As expected, the cortisol responses to both isolation and restraint and barking dogs were greater in nonlactating ewes than that in lactating ewes. Furthermore, there was greater attenuation in the cortisol response to isolation and restraint in lactating ewes with lambs present and able to be suckled than that in lactating ewes with lambs present but unable to be suckled. These findings support our previous research with ewes [4], indicating that the presence of lambs with the opportunity to suck results in maximal attenuation of HPA axis activity in response to isolation and restraint. With respect to barking dogs, both groups of lactating ewes had greater plasma cortisol, but the increase was less in the lactating ewes with lambs present and able to be suckled than the ewes with lambs present and unable to be suckled. This suggests that the HPA axis in lactating ewes will respond to predator stress in the form of barking dogs, albeit to a reduced extent compared with nonlactating ewes. In addition, our data indicate that the presence of lambs and opportunity to suckle result in the greatest reduction in this response. Overall, these findings are supportive of the hypothesis that the extent of attenuation of the HPA axis to stress in lactating ewes is influenced by the type of stressor. In contrast to cortisol, plasma concentrations of oxytocin were not influenced by either stressor in this experiment although, as expected, oxytocin concentrations were greater in lactating than in nonlactating ewes.

The different cortisol responses in lactating ewes to isolation and restraint and barking dogs support the contention that stress responses in lactating females will vary depending on the extent to which behavioral and physiological actions of the dam are required to protect the offspring in a threatening environment. As indicated in the introduction, isolation and restraint of ewes when their lambs remain with them is unlikely to be perceived as posing a major direct threat to the offspring. In contrast, barking dogs are likely to be identified as a greater threat, and this would require physiological and behavioral adaptions to allow the ewe to protect her offspring and herself. Activation of the HPA axis, and other stress systems, will acutely stimulate a range of autonomic, hormonal, and behavioral processes in the ewe to maximize survival [13]. Different responses of the HPA axis to isolation and restraint and barking dogs would have been unlikely because of differences in the intensity of the stressors because both stressors elicit similar maximal concentrations of cortisol in nonlactating ewes [15], [16], and there were no differences in mean concentrations in the present study. Our direct findings with ewes in this study provide support for the indirect findings in lactating rats [8] and rhesus macaques [20] where the HPA axis was stimulated in circumstances where the offspring may have been threatened. Furthermore, our data are in agreement with humans where breastfeeding mothers showed stress responses when there was a perception of a threat to the survival of the mother, and as a consequence, her infant [10]. In this study, there were high plasma concentrations of cortisol in nonlactating ewes before isolation and restraint. The reasons for this are not known, but this has been observed previously in these types of studies [15]. This was taken into consideration with the statistical analysis by considering all sources of variation and does not detract from the findings. Importantly, our work has extended the circumstantial interpretations of others [8], [10], [20] in different species to provide conclusive evidence that when a stressor threatens the dam and her offspring, mechanisms are evoked that overcome, or diminish, the stress hyporesponsive state of lactation, at least to some extent. The mechanisms, and the stimulatory cues, have not been identified.

The specific mechanisms for divergent activation of the HPA axis in lactating ewes with lambs present and able to suck in response to isolation and restraint and barking dogs are unknown. Nevertheless, some of the underlying mechanisms that cause lactation to be a generally stress hyporesponsive condition have been identified. These include reduced synthesis and secretion of CRH and AVP from the PVN because of reduced activity of neural excitatory inputs and increased negative inputs, decreased pituitary responsiveness to CRH and AVP, decreased adrenal responsiveness to ACTH, and altered negative feedback by glucocorticoids (for reviews see [5], [13], [21]). Presence of the offspring provides many cues, such as vocalization, odors, and a range of behaviors that likely include sucking, to stimulate some or all these mechanisms ([4], [19] present study). Oxytocin is released by these cues [5], [13] and has critical reproductive functions such as lactogenesis, galactopoiesis, milk ejection, and parturition [20], [22], [23] and in maternal behavior [24]. Because there is experimental evidence that oxytocin can act centrally to attenuate the activity of the HPA axis, [14] it stands to reason that this nanopeptide could be considered a mediator of the hyporesponsiveness of the HPA axis during lactation. In the present study, we found greater plasma concentrations of oxytocin in lactating than nonlactating ewes, which, as mentioned, was expected, but our data do not provide direct support for a role for oxytocin in influencing the activity of the HPA axis. It could be argued that the greater plasma oxytocin associated with reduced activity of the HPA axis in lactating ewes compared with nonlactating ewes provides a basis for suggesting that oxytocin is a possible mediator of stress hyporesponsiveness. In other words, the oxytocin environment during lactation may have provided the endocrine and neuroendocrine conditions for the HPA axis to be less responsive to stress. Nonetheless, this relationship is correlational and is not unequivocal. Furthermore, there was no increase in oxytocin in response to either stressor in any of the ewes, irrespective of whether they were lactating or not. The latter finding was unexpected because there is evidence that oxytocin secretion will increase in response to some stressors [25]. The type of stressor may be important in this regard although this has not been determined.

The reasons for the lack of oxytocin response to stress in the present study are unknown. One possibility is that the sampling regimen was insufficient to detect small increases in oxytocin. Furthermore, we were likely to be measuring mostly peripheral oxytocin, secreted from the posterior pituitary, whereas it may well be central oxytocin that is predominantly responsible for attenuating effects on the activity of the HPA axis [19], [26]. There is evidence that central oxytocin is involved in attenuation of the HPA axis [5], and we have shown that oxytocinergic neurons in the PVN are well placed to influence CRH and AVP neurons in sheep [19]. The influence of peripheral oxytocin to the stress-induced activation of the HPA axis is not well understood. Although given that oxytocin decreases pituitary responsiveness to CRH and AVP [27], it is generally accepted that peripheral oxytocin is able to influence the activity of the HPA axis. There has been dispute about whether or not oxytocin is able to cross the blood brain barrier, but there now appears to be acceptance that it can [28], [29], [30]. Thus, there is potential for peripheral oxytocin to influence the activity of the HPA axis by acting within the brain as well on the anterior pituitary gland and, perhaps, the adrenal glands. Whatever the source and sites of actions, the weight of evidence is that oxytocin is a likely mediator of stress hyporesponsiveness. Nonetheless, systematic studies are required to establish the relative contributions of central and peripheral oxytocin to influencing the activity of the HPA axis and sites and mechanisms of action.

Our findings are directly applicable to sheep and most likely other domestic livestock species because they demonstrate that stress responses for ewes likely differ depending on whether they are lactating or not and the type of stressor. In terms of animal welfare, this means that separate or tailored protocols that address management during each stage of production may be valuable. Indeed, practices that may evoke a stress response in a nonlactating ewe may not evoke the same stress response in a lactating ewe, and our data are good evidence of this. In addition, our data suggest that anything that a ewe perceives as a threat to her lambs will evoke a greater stress response during lactation than a stressor that is not perceived as a threat to her lambs. An evaluation of other predator stressors similar to that of a barking dog, like the presence of a human, may be valuable in understanding stress responses and animal welfare in production animals.

## 5 Conclusion

In conclusion, the response of the HPA axis to stress is attenuated during lactation in sheep, but the extent of the attenuation is influenced by the opportunity to suckle lambs and the type of stressor. The attenuation of the HPA axis is maximized when there is an opportunity to suckle lambs. Furthermore, stressors that potentially threaten the wellbeing of the ewe and her lamb will evoke greater responses of the HPA axis. Thus, under conditions where a stress response is required for survival, or to maximize wellbeing, there is less hyporesponsiveness of the HPA axis than in less threatening circumstances. The mechanisms are unknown, including the role of oxytocin. We have shown here that lactating ewes have greater circulating levels of oxytocin and lower levels of cortisol in response to stress than nonlactating ewes, but there were no changes in plasma oxytocin in response to stress. There is now a solid platform on which to build further research to understand, and potentially manipulate, the mechanisms involved in attenuation of the HPA axis during lactation.

## Figures and Tables

**Table 1:** Mean (± standard error of the mean) plasma concentrations of cortisol (ng/mL) for nonlactating ewes, lactating ewes with lambs present but unable to suckle, and lactating ewes with lambs present and able to suckle (groups) 1 h before (prestress) and during the 4 h (stress) of each stressor (isolation and restraint, barking dogs).

Groups	Stressor	Prestress cortisol (ng/mL)	Stress cortisol (ng/mL)	Proportional increase^c^
Nonlactating ewes	Isolation and restraint	26.4 ± 4.8^a^	38.4 ± 7.8^b^	1.45^x^
Barking dogs^d^	9.3 ± 3.0^a^	47.3 ± 10.7^b^	5.11^y^
Lactating ewes with lambs present but unable to be suckled	Isolation and restraint	9.5 ± 2.1^a^	15.1 ± 2.9^b^	1.59^x^
Barking dogs	9.5 ± 1.8^a^	23.4 ± 4.1^b^	2.45^y^
Lactating ewes with lambs present and able to be suckled	Isolation and restraint	9.1 ± 4.3^a^	9.8 ± 1.6^a^	1.07^x^
Barking dogs	6.0 ± 2.5^a^	13.0 ± 2.4^b^	2.16^y^

Significant differences (*P* < 0.01) between prestress and stress for each stressor are illustrated by the different superscripts ^a,b^. Significant differences (*P* < 0.05) in proportional increase in mean plasma concentrations of cortisol are illustrated by the different superscripts ^x,y^.

c Proportional increase in plasma concentrations of cortisol from prestress to stress, calculated as the ratio of prestress to stress.

d Three dogs were introduced to vicinity of the pens containing sheep and barked continuously for 5 min every hour during the 4 h.
